# Design and determination of the psychometric properties of a fear of childbirth tool for Iranian adolescent mothers: An exploratory sequential mixed method study protocol

**DOI:** 10.1371/journal.pone.0320616

**Published:** 2025-04-22

**Authors:** Bibi Leila Hoseini, Abbas Ebadi, Ali Mashhadi, Mohammad Hassan Rakhshani, Raheleh Babazadeh

**Affiliations:** 1 Department of midwifery, School of Nursing and Midwifery, Student Research Committee, Mashhad University of Medical Sciences, Mashhad, Iran; 2 Nursing Care Research Center, Clinical Sciences institute, Baqiyatallah University of Medical Sciences, Tehran, Iran; 3 Department of Psychology, Ferdowsi University of Mashhad (FUM), Mashhad, Iran; 4 Department of Biostatistics & Epidemiology, School of Public Health, Sabzevar University of Medical Sciences, Sabzevar, Iran; 5 Nursing and Midwifery care research center, Mashhad University of Medical Sciences, Mashhad, Iran; 6 Department of midwifery, School of Nursing and Midwifery, Mashhad University of Medical Sciences, Mashhad, Iran; Instituto Nacional de Salud Publica, Mexico

## Abstract

**Introduction:**

Fear of childbirth (FOC) is a crucial health issue during pregnancy and childbirth, especially among adolescent mothers. Despite emotional, physical and psychological complications of fear of childbirth, perinatal fear and anxiety have been neglected in practice. One reason is the lack of appropriate tools for timely measurement of fear in different target groups. Therefore, researchers have decided to design a fear of childbirth tool and determine its psychometric properties for Iranian teenage mothers on the basis of the cognitive vulnerability model.

**Methods and analysis:**

This exploratory sequential mixed method study will be conducted in two qualitative and quantitative phases. In the qualitative phase, qualitative content analysis is performed on the basis of the cognitive vulnerability model (CVM) via interviews with pregnant adolescent women and up to 6 months after delivery. The data are analyzed according to Elo and Kyngas (2008) in three steps: preparation, organization and reporting. The quantitative phase of the study will be performed in two parts: tool design and psychometrics. We develop the FOC tool according to Waltz et al. (2017). The first step is to select a conceptual model that will be used from the results of the first phase (qualitative method). The next steps include explicating the objectives for the measure, developing the blueprint, and constructing the measure. The psychometrics of the tool will be determined via validity, including content, face and construct validity (exploratory and confirmatory factor analysis), reliability (internal consistency and relative reliability) and responsiveness.

**Conclusion:**

Since there is no specific FOC tool for adolescents and the probability of this group being more vulnerable to childbirth, it is necessary to develop a valid tool specifically for adolescents to assess fear of childbirth.

## Introduction

Fear of childbirth (FOC) is one of the most common problems that women experience during pregnancy and childbirth [1]. It has been defined as a health issue in pregnancy in relation to anxiety problems or a phobia that interferes with daily activities and well-being. This may lead to physical problems, a lack of concentration, a nightmare and a need for a cesarean section (CS) [2–4]. Fear of childbirth is more common in adolescents than in adults. According to one study, 75% of teenagers were afraid of childbirth; among them, 37.4% had severe fear of childbirth [5]. In addition, Ölmez et al. (2021) showed that the prevalence of fear of severe childbirth in Turkish teenagers was 21.8% according to the Wijma tool, which is a high prevalence [4]. Adolescent pregnancy is a major health issue in the 21st century [6]. There is wide variation in the total fertility rate (TFR) of adolescents worldwide. For example, adolescents’ TFR in Southeast Asia (2018) was 33.6, whereas it varied from 0.3 in the Democratic Republic of Korea to 83 in Bangladesh [7]. The fertility rate of teenagers in Iran was 40.8 per thousand in 2020 [8]. Adolescent pregnancy is associated with significant emotional, medical, social and economic outcomes for mothers, children, parents, and society as a whole. The fact that adolescents have not reached sufficient physical, emotional, cognitive and social development makes control against fear of childbirth a more sensitive and important issue in this age group [5,9]. Furthermore, their double transition period, i.e., pregnancy and adolescence, can increase the level of stress and physical and psychological vulnerability [4,10].

Vulnerability is defined as a person’s perception of being exposed to internal or external dangers and not having control or insufficient control to provide a sense of safety. How a person comprehends a stimulus is suggested to be crucial in assigning fear in relation to the stimulus. Perceiving the stimulus as dangerous, disgusting, uncontrollable and unpredictable creates a schema of vulnerability, referred to as the cognitive vulnerability model (CVM) [11].

The experience of vulnerability unique to childbirth is proposed with the woman’s exposure to this contextual framework and her perception of childbirth. Women are vulnerable socially, psychologically and physically during childbirth. The women’s transition from pregnancy to motherhood and acceptance of a new role cause women to be exposed to social harm and injuries. Being exposed to physical injuries, feeling unstable and not meeting their expectations cause physical and psychological vulnerability [12]. Furthermore, according to several studies, women’s perceptions of childbirth are uncontrollable [12, 13], unpredictable [14, 15], harmful and dangerous for the mother and the fetus [15–19] and disgusting [12,20]. Thus, it seems that childbirth also creates a schema of vulnerability, and this model can be used to explain the concept of fear of childbirth in teenage mothers.

FOC has many adverse life-long outcomes for women’s health and leads to poor relationships with their infant [2,21–23], partner and family [2,22]. It results in the inability to cope with childbirth, unwillingness to become pregnant, termination of pregnancy, increased perception of pain at birth, and obstetric consequences, including long labor, increased risk of emergency cesarean section, low childbirth satisfaction, and postpartum depression [19,21], in addition to posttraumatic stress disorder (PTSD) [19,24]. Moreover, it increases elective CS among women to escape from vaginal delivery [2,24].

Given the increased prevalence of adolescent pregnancy, particularly in developing countries, and the aforementioned risks, it is necessary to prevent mental and physical health challenges in this vulnerable population (4). Therefore, developing a valid tool to measure FOC among adolescents as a high-risk group that is influenced by adverse consequences of FOC, especially reproductive health, is necessary. Yousefi et al. (2021) addressed FOC among nulliparous adolescents in the third trimester of pregnancy in a qualitative study [25]. On the other hand, according to the literature review, there are many tools for FOC assessment in adults, including one-item tools such as those implemented by Storksen et al. (2012) [26], for the first time, tools with 53 items, e.g., Melender et al.‘s tool (2002) [27]. The evidence has shown that most of these measurements do not have enough psychometric properties, so they are not valid enough to use [28]. More importantly, they are not developed specifically for adolescent mothers, who are more vulnerable.

To the best of our knowledge, we have not found any fear of childbirth tools in the context of Iran, specifically among adolescent mothers worldwide. Furthermore, no study has implemented a cognitive vulnerability model for fear of childbirth. Moreover, the content of tools should be extracted from the target population, i.e., adolescent mothers, to achieve complete perceptions of FOC. Thus, researchers have aimed to design a fear of childbirth tool for Iranian adolescent mothers on the basis of the CVM and determination of its psychometric properties.

## Materials and methods

### Study aims

Design and determination of the psychometric properties of a fear-of-childbirth tool for Iranian adolescent mothers.

### The objectives of the qualitative phase

- Clarifying the perception and experience of fear of childbirth from Iranian adolescent mothers’ point of view
- Clarifying the physiological response of Iranian adolescent mothers to the fear of childbirth
- Clarifying the behavioral response of Iranian adolescent mothers to the fear of childbirth
- Clarifying the cognitive response of Iranian adolescent mothers to the fear of childbirth
- Clarifying the emotional response of Iranian adolescent mothers to the fear of childbirth

### The objectives of the quantitative phase

- Design of the fear of childbirth tool for adolescent mothers
- Determination of the validity of the fear of childbirth tool for adolescent mothers
- Determination of the reliability of the fear of childbirth tool for adolescent mothers

### Study design

The present study is an exploratory sequential mixed methods study. The mixed method studies are a design with a pragmatic paradigm in which the researcher collects data via two quantitative and qualitative approaches, analyzes them and then integrates them with each other [29]. The time order of the present research will be sequential, containing qualitative and then quantitative steps. Therefore, the researcher explains the perceptions and experiences of the participants regarding their fear of childbirth via the first phase, i.e., the qualitative content analysis method. In the quantitative phase, the main items of the tool for the fear of childbirth tool for adolescent mothers will be developed on the basis of the data obtained from the first phase of the study. The researcher subsequently determines the psychometric properties of this tool.

#### Phase 1: The qualitative phase.

Qualitative content analysis is applied in many health studies, especially exploratory designs or studies, with the aim of describing important phenomena for a certain group of people. Qualitative content analysis is divided into three conventional, directed and cumulative approaches [30]. The method used depends on the aim of the study. In the directed approach, the theory already exists [31]. Since the cognitive vulnerability model (CVM) is used to conceptualize fear acquisition, we aim to confirm this theory. A theory that already exists helps to focus on the research questions. It determines the initial coding scheme and the relationship between the codes, which indicates the deductive classification. Deductive content analysis is applied when the basis of the analysis is previous knowledge and when theory testing is the aim of the study [31].

**Cognitive vulnerability model (CVM):** As mentioned before, the perception of a stimulus as dangerous, disgusting, uncontrollable and unpredictable creates a cognitive vulnerability model (CVM) [11]. Some specific phobias, such as spider and dental fear, have been investigated with the cognitive vulnerability model [11,32]. According to this model, individual differences such as biological characteristics as well as learning experiences such as exposure to similar stimuli affect the vulnerability schema. Immediately after the activation of the vulnerability schema, two parallel processes involving an automatic affective reaction and then a general cognitive evaluation of the stimulus occur. These two processes lead to cognitive, physiological and behavioral responses in addition to a quick emotional response to the fear stimulus [32].

- **Emotional response**: This includes negative and positive emotions. Negative emotions such as being sad, fearful, terrified, and worried. Positive emotions such as happiness, relief, and peace [32].
- **Physiological response:** According to the CVM, subdomains of the vasovagal response and specific physiological responses are considered physiological responses [32].
- **Behavioral response:** The subdomains of behavioral response in the CVM model include avoidance and escape [32].
- **Cognitive response:** The subdomains of the cognitive response based on the CVM model are preoccupation and catastrophizing [32].

**Settings:** The sampling environment includes health centers and hospitals as well as the agreed upon location with the participants and researcher in the cities of Mashhad and Sabzevar (east north of Iran), which makes it possible to access participants and gather information.

**Participants:** The participants are adolescent pregnant women aged 10–19 years, during pregnancy or up to six months after giving birth who are speaking Farsi fluently. The mothers not willing to complete the interview will be excluded. If needed, health service providers (including midwives, reproductive health specialists and gynecologists) will also be interviewed.

**Sampling:** In the qualitative phase of the present study, purposive sampling of the target population, including pregnant or postpartum adolescents, is performed, with maximum variation in maternal age, number of childbirths, gestational age (GA), time of delivery, and economic, cultural and social status among adolescent mothers. Sampling will continue to acquire data saturation.

**Data collection:** The research project approved by the Research Council and the Ethics Committee of Mashhad University of Medical Sciences with ethical code number IR.MUMS.NURSE.REC.1402.016. The recruitment of the eligible participants for interview has started from 2023-12-22 and will be ended 2024-12-30 in the cities of Mashhad and Sabzevar.

First, a general explanation related to the objectives of the research is given to the participants, and written informed consent is obtained. Mothers are asked about the appropriate location for the interview. They were informed that their voice will be recorded during the interview. The interviews will last about 30-60 minutes. Additionally, they are assured that their voices will not be published elsewhere. Their information is registered via a demographic questionnaire. Semi-structured interviews will be conducted by a well-informed interviewer about the target population’s experiences in a quiet and appropriate environment. The research team is a skilled moderator to standardize the process among all participants and to ensure that the gathered information is relevant to tool development. The interviewer will also take notes during the interviews. The interviews will be conducted with an interview guide including descriptive questions such as “What is your gestational age? “/”How long has it been since you gave birth?”. It also contains structural questions, including “Imagine yourself in the delivery room, how do you feel?” (Emotional response), “When you have fear of childbirth, can you explain what changes happen in your body? (Physiological response)”, “At that moment when you thought about giving birth and got scared, what thought came to your mind? “Cognitive response-- Preoccupation)”, “What is the worst thing that can happen during childbirth that scares you? (Cognitive response - catastrophizing)”, “What do you do to reduce the fear of childbirth? What do you decide to do for your birth? (Behavioral response-avoidance and safety behaviors)”.

In addition, exploratory questions such as “Could you explain more?” or “What did you mean by this sentence?” will be asked. Finally, to ensure the completeness of the interviews, questions such as “Is there anything else that you have not said? “, “Do you think there is a question that has not been asked?”. Immediately after the interviews will be transcribed verbatim on a word file.

**Reflexivity:** The interviews are conducted by a researcher who had passed academic courses related to the methods of qualitative research besides a training workshop entitled how to conduct the interviews in qualitative research. In addition, initial interviews were ignored until necessary skill was acquired to interview under supervision of an expert researcher. The qualitative phase of the study is supervised by a professional researcher who is a qualitative research instructor and has extensive experience in conducting (including interviewing, coding and analyzing the data) and supervising qualitative researches and tool development. On the other hand, the researchers are experienced in interacting with women in pregnancy and postpartum period especially in childbirth. Moreover, the interviews and data analysis are followed and peer-reviewed step by step by three reviewers who are experienced in qualitative research and childbirth.

**Data analysis:** MaxQDA software version 2020 is used for data management in qualitative data analysis. The data analysis of the present study is performed via the analysis method described by Elo and Kyngas (2008), using deductive content analysis because of the presence of previous knowledge (cognitive vulnerability model) with an unconstrained matrix to develop more concrete and understandable concepts. When an unconstrained matrix is used, different categories can be created within its bounds. In addition, earlier FOC tools can be involved using unconstrained matrix of analysis. According to Elo and Kyngas (2008), in the process of qualitative content analysis, there are three phases: 1) the preparation phase, which includes choosing the analysis unit and making sense of the data and whole topic; 2) the organizing phase, which includes developing analysis matrices, extracting data from the content on the basis of codes; classification; categorization; and abstraction, which involves two coders who are thoroughly trained. 3) The reporting phase (reporting the analysis process and results), which includes summarizing, presenting the model, conceptual system, and conceptual map [31].

**Trustworthiness:** Lincoln and Goba’s (1985) trustworthiness criteria include credibility, dependability, confirmability, transferability, and authenticity [33]. The credibility of the present study increases through being the researcher in the field of sampling and being immersed in the data by repeatedly reviewing them, as well as using data triangulation (using data from different times, spaces, and people) and peer review by supervising expert researchers in the field of reproductive health, specifically childbirth and psychology. To increase dependability, the researcher elaborates the study schedule, tables and appendices that explain the categorization process. To increase confirmability, all the steps will be written clearly so that other researchers can also follow the data. Moreover, the text of the interviews and data analysis steps will be given to some reviewers who are experienced in qualitative research to confirm the research data. Continuous monitoring of the research from the beginning to the end will also be performed to increase the objectivity and homogeneity of the data. To increase transferability, we describe the context, environment, sampling method and participants’ characteristics, as well as the data collection and analysis process in detail. To increase authenticity, we will cite quotes from different participants without mentioning their names in the report of the research.

#### Phase 2: The quantitative phase.

**Settings:** The sampling environment includes health centers and hospitals as well as the agreed upon location with the participants and researcher in the cities of Mashhad and Sabzevar (east north of Iran), which makes it possible to access participants and gather information.

**Participants:** The participants are adolescent pregnant women aged 10–19 years, during pregnancy or up to six months after giving birth who are able to read and write. The mothers who will not complete the questionnaires will be excluded.

**Data collection:** The recruitment of the eligible participants in the quantitative phase will be started from 2025-1-30 and will be ended 2025-12-30. First, a general explanation related to the objectives of the research is given to the participants, and written informed consent is obtained.

**Part 1: Developing the tool:** According to Waltz et al. (2017), the essential steps in the design of a tool are as follows [34]:

1. **Selecting a conceptual model:** To delineate the health care or nursing aspects of the measurement process.
2. **Explicating the objectives for the measure**: This step clarifies the purposes for the measurement.
3. **Developing a blueprint**: In this step we develop a blueprint to make the specific scope and emphasis of the measure. Further, to enrich the initial pool, the researcher will review articles related to the fear of childbirth in teenage women systematically.
4. **Constructing the measure**: The type of measure as a function of the conceptual model and subsequent operational definition of key variables to be measured. Every measure also contains directions for administration, a set of items, and directions for obtaining and interpreting scores.

**Part 2: Psychometric properties of the tool:** To determine the psychometric properties of the tool, validity, reliability and responsiveness are examined. The analysis will be performed via SPSS version 2022 and LISREL software.

**Validity:** Validity has three main parts: content validity, formal validity, and structural validity.

**Face validity:** To determine face validity, quantitative and qualitative methods are used. To qualitatively determine face validity, ten teenage mothers, will be interviewed face to face. The level of difficulty, relevancy and ambiguity of the tool is subsequently assessed. To quantitatively determine facial validity, after the items are revised on the basis of people’s opinions via the qualitative method, the quantitative method of “item impact” is used to reduce and eliminate irrelevant items and determine the importance of each item. The 5-point Likert scale included 5 (completely important), 4 (somewhat important), 3 (moderately important), 2 (slightly important), and 1 (not important at all). The procedure will be as follows: 10 teenage mothers will be asked to assess the items and select one option.

Impact item score = frequency (%) × importance

Frequency (%): The number of people who scored items 4 and 5

Importance: The average score of importance based on the Likert scale

If the impact score is greater than 1.5, the item is suitable for further analysis and will be retained [35].

**Content validity**:

**A.Qualitative content validity:** Ten relevant experts (in the fields of midwifery, reproductive health, gynecology, psychology and nursing) are requested to provide written revisions of the tool. The items are assessed for qualitative content validity, including grammar, wording, item allocation and proper scoring.

**B.Quantitative content validity:** The content validity ratio (CVR) and content validity index (CVI) are used to quantify the content validity of an assessment tool as evaluated by clinical experts.

The CVR is calculated for each item with a Likert scale including 0 (not necessary), 1 (useful), or 3 (necessary). The numeric value of the CVR is determined by the Lawshe Table. The formula of the CVR is as follows:


$$CVR=\frac{\left[Ne-\left(N/2\right)\right]}{\left(N/2\right)}.$$

Ne: The number of panelists indicating “essential”

N: The total number of panelists.

In the present study, the preliminary tool will be sent to approximately 10 experts. The result obtained after the calculation is compared with the standard in the Lawshe table according to the number of experts in the present study. A larger number obtained from the table indicates that the item is necessary and important in this tool with statistical significance (P < 0.05).

The CVI consists of the evaluation of each item by each expert panel member. Items are evaluated on the basis of their relevance, clarity, simplicity, and specificity. However, some experts believe that when calculating CVI, “relevance” is more important. Therefore, the panel members evaluate each item via a four-point Likert scale (1 =  irrelevant to 4 =  very relevant). Finally, the CVI is expressed as an item-content validity index (I-CVI). To calculate the I-CVI, the number of experts who scored each item 3 (completely relevant) or 4 (very relevant) is divided by the total number of experts who participated in the evaluation. If I-CVI < 0.7, the item should be removed; I-CVI = 0.7--0.79 needs revision and correction; and I-CVI >  0.79 is considered appropriate.

The use of the kappa statistic instead of the CVI is recommended because of the inclusion of chance agreement. A modified kappa value was calculated, the kappa designating agreement of relevance, via the following formula:


$$K=\frac{ICV1-Pc}{1-Pc};Pc=\left[\frac{N!}{A!\left(N-A\right)!}\right]\times 0.5^{N}$$

N: the number of evaluators; A: the number of agreements regarding the relevance of the item; Pc: the chance agreement ratio.

The modified kappa coefficient is interpreted as follows: K = 0.4--0.59 indicates poor agreement, K = 0.6--0.74 indicates good agreement, and K ≥  0.74 indicates very good agreement [35].

**Item analysis:** An item analysis was performed on approximately 50 teenage mothers to determine the initial reliability of the questionnaire and determine the items that affect its reliability. We will use loop method for this purpose. The correlation of the items with each other and with the total score will be determined; the items with a correlation ≥ 0.8 with each other (merged) or with a correlation < 0.3 with the total score (corrected item total correlation) will be removed. Additionally, if an item does not have a correlation coefficient greater than 0.3 with at least one other item from the questionnaire, it should be removed [36].

**Construct validity:** To determine construct validity, we use exploratory factor analysis (EFA) and confirmatory factor analysis (CFA). To do factor analysis, we will use the linear approach (Pearson correlation matrix).

**Exploratory factor analysis (EFA):** Items that are closely related to each other are grouped into one factor via EFA. Two main goals of exploratory factor analysis are data reduction and the explanation and clarification of the theoretical structure. The three main steps of factor analysis are as follows: 1. The correlation matrix of all variables is calculated 2. Extracting primary factors 3. Rotation of the extracted factors. [36,37].

We will use Bartlett’s test of sphericity to determine the operability of the data, and also the Kaiser‒Meyer‒Olkin (KMO) index to determine the adequacy of the data. The basis of KMO is that if variables share common factors, then partial correlations between pairs of variables should be small when the effects of other variables are controlled. The KMO index ranges 0-1. The KMO ≥ 0.8 supports the use of factor analysis for the data. Bartlett’s test of sphericity is used to evaluate whether a correlation matrix is suitable for factor analysis. When Bartlett’s test is significant, it is the indicator of operability of the data [38].

There are several methods to extract data. Principal factors (PF) and maximum likelihood (ML) are two of the most popular estimation methods in EFA. One of the most advantages of the ML estimation method is that how closely do the correlations among the indicators predicted by the factor analysis parameters approximate the relationships observed in the input correlation matrix. It is a very useful feature to determine the proper number of factors. But, ML estimation needs the assumption of multivariate normal distribution of the variables. The other potential disadvantage of ML estimation is its tendency to produce “improper solutions.” An improper solution exists when a factor model does not converge on a final set of parameter estimates, or makes an “out of range” estimate like an indicator with a communality above 1.0. However, PF has the main advantages including being free of distributional assumptions and also being less prone to improper solutions rather than ML. PF does not provide goodness-of-fit indices helpful in determining the suitability of the factor model and the number of latent variables. Therefore, PF might be preferable in cases where evident non-normality is seen in the observed measures or perhaps when ML estimation produces an improper solution [39].

To determine the number of factors, the following rules are used. Kaiser’s rule, which requires eigenvalues > 1, minimum variance of each factor 5% and the variance > 50% for the entire tool, to be supplemented with Scree plot, by using visual examination.

There are two main categories of rotation options including oblique rotation, which allows for small-to- moderate correlation of factors, and orthogonal rotation, which assumes that the factors are uncorrelated [35,38].

**Confirmatory Factor Analysis (CFA):** Validation of the number of factors or subsets of the tool and measurement of equality between comparison groups is performed through confirmatory factor analysis (CFA) [37]. The steps of how to perform CFA are as follows: 1. Model specification 2. Recognition 3. Model estimation 4. Model evaluation 5. Model Modification [35].

Table 1 shows the acceptable thresholds for the goodness of fit indices and the results of CFA.

**Table 1:** The acceptable thresholds for the goodness of fit indices and the results of CFA [35].

Acceptable range	Fitting indices
0.05 <	Chi-squared *P*-value (χ^*2*^ *P*-value)
Good < 0.08, medium < 0.08- 0.1, weak < 0.1	Root Mean Square Error of Approximation (RMSEA)
< 0.1	Standardized Root Mean Square Residual (SRMR)
<0.5	Parsimonious Normed Fit Index (PNFI)
<0.5	Parsimonious Comparative Fit Index (PCFI)
<0.5	for close fit of the population RMSEA (PCLOSE(
<0.9	Normed Fit Index (NFI)
<0.8	Adjusted Goodness of Fit Index (AGFI)
<0.9	Goodness of Fit Index (GFI (
<0.9	Relative Fit Index (RFI(
<0.9	Tucker-Lewis Index (TLI)
<0.9	Comparative of Fit Index (CFI)
Good < 3 and Acceptable < 5	Minimum Discrepancy Function by Degrees of Freedom divided (CMIN/DF)

In general, the indicators used to examine the goodness of fit are divided into three general categories:

Absolute fit: GFI, SRMR, RMSEA, Chi-squared

Comparative fit: CFI, RFI, NFI, TLI

Parsimonnious fit: PCFI, PNFI, AGFI [35]

We will test the convergent validity of our tool by comparing the relationship between the FOC and the W-DEQ with the Pearson correlation coefficient. We will use W-DEQ which was validated in Iran [40].

**Sample size:** To determine sample size in factor analysis, there are some rules including the participants per variable ratio (10 to one) and the variable to expected factors ratio (minimally 3 to 1). The other studies mention the factors affecting the sample size such as common variance estimation, factor loadings and also overdetermination [38]. Based on the COnsensus-based Standards for the selection of health status Measurement INstruments (COSMIN) checklist, a very good sample size for factor analysis is a sample size seven times the number of items[41]. In the present research, the sample size will be considered seven times the number of items for EFA and also CFA. In other words, we need a total sample of 420 participants for a 30-item tool. According to Lorenzo‑Seva (2022), a split method aims to produce equivalent subsamples [42]. So, we will split the sample randomly into two sub-sample for EFA and CFA.

**Reliability:** The reliability of this tool is determined through internal consistency and relative reliability. The internal consistency will be determined via Cronbach’s alpha, and Omega internal consistency coefficient. The relative reliability in this study should be assessed at a fixed period for each person, i.e., pregnancy, or postpartum, by 50 teenage mothers [36] at two-week intervals by obtaining an intra-class correlation (ICC) for the items.

**Responsiveness:** The formula for determining responsiveness is as follows:


$$SEM_{agreement}=SD\times \surd \left(1-ICC_{agreement}\right)$$

MDC=1.96×√2×SEM

SEM: standard error of measurement; MDC: minimal detectable change; SD: standard deviation; ICC: intraclass correlation coefficient

## Discussion

The present study aims to design a fear of childbirth tool for Iranian adolescent mothers and determine its psychometric properties. Statistics show that the adolescent population, child marriage rate and fertility rate are rather high, particularly in underdeveloped countries. Fear of childbirth has long-term consequences, especially for adolescents. As mentioned above, adolescents have not completely developed in terms of physical, emotional, cognitive and social aspects in comparison with adults. Several neurobiological models have been designed to account for many affective, cognitive and behavioral changes observed in adolescence. These models suggest that as the prefrontal cortex (PFC) is a late-maturing structure, the connections between the PFC and sub-cortical structures, which develop more rapidly than the PFC does, do not develop until early adulthood or late adolescence. Therefore, the PFC does not have sufficient connections to inhibit behaviors that are mediated by subcortical structures until adulthood and later. This inconsistency results in an imbalance in activity between emotional and reward-driven behaviors due to the low level of connection between these neural regions [43]. As a result, the different development of adolescents from that of adults may lead to different perceptions and experiences of childbirth. Therefore, adolescent mothers have a less positive understanding of birth experience than adult mothers do [44]. However, the only study that has assessed FOC among adolescent mothers is Yousefi et al. (2021) through content analysis. They identified two main categories, namely, fear of childbirth and strategies to address it, in the form of a content analysis study using a conventional method [25].

However, there are educational and psychological approaches to minimize fear of childbirth and adverse consequences, and the key point in managing women experiencing FOC is the precise assessment [19]. There are several scales for assessing fear of childbirth, mostly in adults, such as the fear-of-delivery questionnaire (FDQ) [45], delivery fear scale (DFS) [46], birth anticipation scale (BAS) [47], childbirth fear questionnaire (CFQ) [19], childbirth fear scale (CFS) [48], and fear of childbirth tool by Prelog et al. (2019) [49]. Some FOC tools contain one or two items, including the unnamed tools by Waldenström et al. (2006) [50] and Laursen et al. (2008) [51] and the Fear of Birth Scale (FOBS) [18], visual analog scale (VAS) [52] and numeric rating scale (NRS) [26]. It seems that scales with one or two items do not evaluate FOC in a stable manner [19]. The respondents of the other scales, which contain more items, are also adult women older than 18 or 19 years. Among them, only four measurements were derived from the target population [15,16,53,54]. Three of them involved semi -structured interviews, including the Slade–Pais Expectations of Childbirth Scale (SPECS), the Fear of Childbirth Questionnaire (FCQ), an unnamed tool by Melender et al. (2002) [15,53,54], and grounded theory, i.e., an unnamed tool developed by Eriksson et al. (2005) [16]. The content of all these tools was derived via interviews with pregnant adult women older than 18 years. According to the experts, the content of the tools should be extracted directly from the target population (adolescent mothers) for the issues covered by the tool and the phrasing of the items [55]. The W-DEQ is the most commonly used scale to assess FOC, with several translations worldwide. The content of this scale originated from qualitative research with two researchers with clinical experience related to women who experienced fear of childbirth instead of pregnant or postpartum women [56]. Measures with items originating from experts’ views or the literature review contain professional views of the construct instead of potential respondents’ experience [55]. The W-DEQ assesses many emotions in labor and delivery. However, it seems that it does not contain some dimensions of FOC, such as physiological reactions to fear, such as hyperventilation, tachycardia and sweating.

The present tool can be used by health service providers to objectively measure the fear of childbirth in adolescent mothers. In this way, we can provide care services, counseling or referral services. On the other hand, the findings of this research can be used to promote future studies in line with the intervention programs needed for this group of mothers.

The strength of the present study is that we will extract the content of the tool through interviews with the target population, i.e., Iranian adolescent mothers. If the content of the tool is derived from the respondents, it is ensured that appropriate items are included. On the other hand, since the participants are Iranian women, the generalizability of this tool may be limited. However, we will consider the maximum variation to minimize this limitation.

## Abbreviations

FOC: Fear of childbirth
GA: Gestational age
CS: Cesarean section
TFR: Total fertility rate
CVM: Cognitive Vulnerability Model
PTSD: Posttraumatic stress disorder
CVR: Content Validity Ratio
CVI: Content Validity Index
I-CVI: Item-content validity index
S-CVI: Scale-content validity index
EFA: Exploratory Factor Analysis
CFA: Confirmatory Factor Analysis
PFC: The Prefrontal Cortex
W-DEQ: Wijma Delivery Expectancy/Experience Questionnaire
FCQ: Fear of Childbirth Questionnaire
FDQ: Fear-of-delivery questionnaire
DFS: : Delivery Fear Scale
VAS: Visual analog scale
FOBS: Fear of Birth Scale
NRS: Numeric rating scale
BAS: Birth Anticipation Scale
SPECS: Slade–Pais Expectations of Childbirth Scale
CFQ: Childbirth Fear Questionnaire
CFS: Childbirth Fear Scale
RMSEA: Root Mean Square Error of Approximation
SRMR: Standardized Root Mean Square Residual
PNFI: Parsimonious Normed Fit Index
PCFI: Parsimonious Comparative Fit Index
PCLOSE: for close fit of the population RMSEA
NFI: Normed Fit Index
AGFI: Adjusted Goodness of Fit Index
GFI: : Goodness of Fit Index
RFI: Relative Fit Index
TLI: Tucker-Lewis Index
CFI: Comparative of Fit Index
CMIN/DF: Minimum Discrepancy Function by Degrees of Freedom divided
